# Polyphenolic Composition and Antioxidant Activity of Aqueous and Ethanolic Extracts from *Uncaria tomentosa* Bark and Leaves

**DOI:** 10.3390/antiox7050065

**Published:** 2018-05-11

**Authors:** Mirtha Navarro-Hoyos, Diego Alvarado-Corella, Ileana Moreira-Gonzalez, Elizabeth Arnaez-Serrano, Maria Monagas-Juan

**Affiliations:** 1 Department of Chemistry, University of Costa Rica (UCR), Sede Rodrigo Facio, San Pedro de Montes de Oca, San José 2060, Costa Rica; 2 Department of Biology, University of Costa Rica (UCR), Sede Rodrigo Facio, San Pedro de Montes de Oca, San Jose 2060, Costa Rica; luis.alvaradocorella@ucr.ac.cr; 3 Department of Biology, Technological University of Costa Rica (TEC), Cartago 7050, Costa Rica; imoreira@itcr.ac.cr (I.M.-G.), earnaez@itcr.ac.cr (E.A.-S.); 4 Institute of Food Science Research (CIAL), Spanish National Research Council (CSIC), C/Nicolás Cabrera 9, 28049 Madrid, Spain; mjm@usp.org

**Keywords:** *U. tomentosa*, UPLC-DAD, TQ-ESI/MS, polyphenols, proanthocyanidins, propelargonidins, procyanidins, mass spectrometry, antioxidant

## Abstract

*Uncaria tomentosa* constitutes an important source of secondary metabolites with diverse biological activities mainly attributed until recently to alkaloids and triterpenes. We have previously reported for the first-time the polyphenolic profile of extracts from *U. tomentosa*, using a multi-step process involving organic solvents, as well as their antioxidant capacity, antimicrobial activity on aerial bacteria, and cytotoxicity on cancer cell lines. These promising results prompted the present study using food grade solvents suitable for the elaboration of commercial extracts. We report a detailed study on the polyphenolic composition of aqueous and ethanolic extracts of *U. tomentosa* bark and leaves (*n* = 16), using High Performance Liquid Chromatography coupled with Mass Spectrometry (HPLC-DAD/TQ-ESI-MS). A total of 32 compounds were identified, including hydroxybenzoic and hydroxycinnamic acids, flavan-3-ols monomers, procyanidin dimers and trimers, flavalignans–cinchonains and propelargonidin dimers. Our findings showed that the leaves were the richest source of total phenolics and proanthocyanidins, in particular propelargonidin dimers. Two-way Analysis of Variance (ANOVA) indicated that the contents of procyanidin and propelargonidin dimers were significantly different (*p <* 0.05) in function of the plant part, and leaves extracts showed higher contents. Oxygen Radical Absorbance Capacity (ORAC) and 2,2-diphenyl-1-picrylhidrazyl (DPPH) values indicated higher antioxidant capacity for the leaves (*p <* 0.05). Further, correlation between both methods and procyanidin dimers was found, particularly between ORAC and propelargonidin dimers. Finally, Principal Component Analysis (PCA) analysis results clearly indicated that the leaves are the richest plant part in proanthocyanidins and a very homogenous material, regardless of their origin. Therefore, our findings revealed that both ethanol and water extraction processes are adequate for the elaboration of potential commercial extracts from *U. tomentosa* leaves rich in proanthocyanidins and exhibiting high antioxidant activity.

## 1. Introduction

*Uncaria tomentosa* L., commonly known as cat’s claw, is a creeper vine typical of the rainy tropical forest that belongs to the Rubiaceae family. It is naturally distributed in South America, mainly in Peru and Brazil as well as in Central America, and it is traditionally used as a medicinal plant. Around 50 compounds have been isolated from *U. tomentosa*, from which approximately 35 chemical markers can be considered exclusive to this species, including triterpenes [1], alkaloids [2] and polyphenols [3]. The special interest on this plant is due to the fact that numerous scientific studies report a wide variety of biological activities [4], such as immunomodulatory properties [3], as well as their antioxidant, anti-inflammatory and cardiovascular effects and protective properties against cancer, among others [5]. 

Although originally attributed to alkaloids, recent studies suggest that the health effects derived from *U. tomentosa* could be attributed to a synergistic interaction among different chemical compounds present in this plant [6]. Of particular interest are polyphenols, for which there is strong evidence of having multiple molecular targets, modulating pro-inflammatory gene expression, interacting with phospholipid membranes [7] and modulating pathways related to chronic inflammation and energy metabolism [8]. 

Regarding polyphenols, *U. tomentosa* studies are scarce and mainly focused on a particular subclass, for instance on hydroxycinnamic acids [9,10], flavonols [11], flavan-3-ols [12,13] and cinchonains [14]. Recently, a detailed characterization of *U. tomentosa* leaves, bark, stem and wood has been reported indicating a high flavan-3-ol content, particularly in procyanidin dimers and trimers, propelargonidin dimers and cinchonain-type flavalignans [15] in leaves and bark. Also, assessment of biological activities of *U. tomentosa* proanthocyanidins showed evidence on the relationship between proanthocyanidin contents and antioxidant capacity, antimicrobial effect against *Staphylococcus aureus*, *Enterococcus faecalis* and *Pseudomonas aeruginosa* respiratory pathogens [16], and in vitro cytotoxicity, with high selectivity for AGS gastric and SW620 colon adenocarcinoma cell lines, mainly attributed to the content of propelargonidin dimers [17].

These promising results about the bioactivity of *U. tomentosa* proanthocyanidin extracts and their potential health effects were subject of an industrial patent [18] and prompted the need to further explore the viability of elaborating polyphenolic extracts form *U. tomentosa* using food-grade solvents (i.e., aqueous or ethanolic extracts) with potential use as Dietary Supplements.

Previous studies carried out with *U. tomentosa* aqueous and ethanolic extracts indicated antioxidant activity in vitro [2,19,20] and other biological activities such as important effects on mono- nuclear blood cells [21] with 95% ethanol extracts; however, detailed characterization of the extracts was not performed. Our present study focuses on the detailed phenolic characterization by HPLC-DAD/TQ-ESI-MS of aqueous and ethanolic extracts from both bark and leaves (*n* = 16) of *U. tomentosa* cultivated in Costa Rica, as well as to determine their antioxidant activity by Oxygen Radical Absorbance Capacity (ORAC) and 2,2-diphenyl-1-picrylhidrazyl (DPPH) methods. Finally, statistical analyses to determine the influence of factors, such as plant part and solvents, on the polyphenolic composition of *U. tomentosa* extracts were explored.

## 2. Results and Discussion

### 2.1. Phenolic Yield and Total Phenolic Content in U. tomentosa Extracts

The aqueous and ethanolic extraction methods described in Section 3, allowed to obtain extracts with yields as shown in Table 1. Considering the plant part, leaves exhibited the highest yields independently of the plant location with an overall average of 5.26%, whereas barks showed much lower yields with 2.28% average. For both parts, barks and leaves, ethanolic extraction yields were slightly higher (average: 2.54% and 5.88%, respectively) than those of aqueous extracts (average: 2.01% and 4.64%, respectively). In regards to total polyphenols (TP), leaves showed higher contents than barks and considering the geographical location, *U. tomentosa* leaves from Los Chiles showed the highest TP values in both aqueous and ethanolic solvents (385.6 and 387.6 mg gallic acid equivalents (GAE)/g extract respectively), followed by Asomat (335.0 and 362.4 mg GAE/g extract), whereas Sarapiqui showed the lowest values (218.2 and 321.0 mg GAE/g extract). In the case of barks, Asomat and Sarapiqui ethanolic extracts showed the highest TP values (295.2 and 321.0 mg GAE/g extract, respectively). Similar trends were observed for the flavan-3-ols (PRO) content.

In order to assess the influence of plant part and extraction solvent on TP and PRO content, a two-way Analysis of Variance (ANOVA) was carried out. Results showed significant differences (*p <* 0.05) for both factors, plant part and solvent used, with ethanolic procedure yielding better TP and PRO values for both plant parts. In addition, importantly, findings indicated leaves extracts are richer in both contents than their bark counterparts, which is in agreement with a recent study on *U. tomentosa* phenolic extracts obtained through an extraction and purification method using different organic solvents [15].

### 2.2. UPLC-DAD/TQ-ESI-MS Analysis of U. tomentosa Polyphenolic Extracts

The UPLC-DAD/TQ-ESI-MS analysis was performed in the 16 aqueous and ethanolic extracts from *U. tomentosa*, as described in Section 3. Determination of 32 phenolic compounds was achieved, comprising non-flavonoid polyphenols, including seven hydroxybenzoic acids, namely benzoic, 4-hydroxybenzoic, salicylic, gallic, protocatechuic, syringic and vanillic acids; and four hydroxycinnamic acids, namely caffeic, *p*-coumaric, ferulic and isoferulic acids. Among flavonoid polyphenols, both flavan-3-ols monomers [(+)-catechin and (−)-epicatechin] were found, as well as eight procyanidin dimers, three procyanidin trimers, and four propelargonidin dimers. Also, four flavalignans-cinchonains were identified (Table 2 and Table 3). 

Multiple reaction monitoring (MRM) transitions under set tandem mass spectrometry (MS/MS) parameters were recorded for compounds found in extracts from *U. tomentosa* bark and leaves. For instance, at *m*/*z* 289/245 for flavan-3-ols monomers [(+)-catechin and (−)-epicatechin], at *m*/*z* 577/289 for procyanidin dimers, at *m*/*z* 561/289 for propelargonidin dimers, at *m*/*z* 865/577 for procyanidin trimers and at *m*/*z* 451/341 for flavalignans-cinchonains. As shown in Figure S1 (Supplementary Materials), MS/MS allowed to identify the different procyanidin dimers and propelargonidin dimers. These results constitute the first report, to our knowledge, of propelargonidins presence in *U. tomentosa* ethanolic and aqueous extracts.

**Figure 1:**
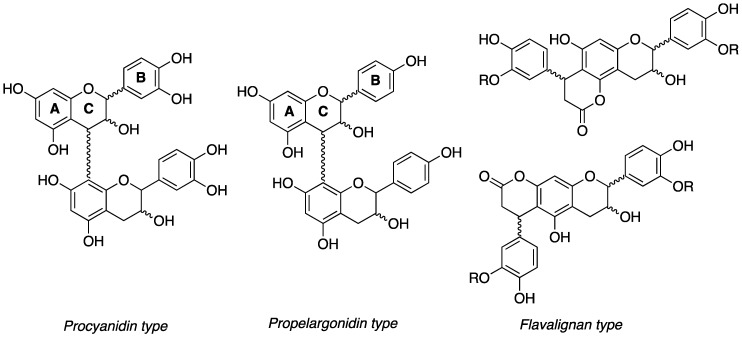
Procyanidin (PC), propelargonidin (PP) and flavalignans (FL) general chemical structures.

In agreement with the TP and PRO determinations, the UPLC analysis revealed that the leaves have higher phenolic content (17,482.3–32,323.3 µg/g of extract) than the barks (5122.0–17,770.2 µg/g of extract). Differences in the distribution of phenolic compounds also occur. For example, in the case of flavan-3-ols (Figure 1) propelargonidin dimer (*Rt* = 4.43 min) (3067.8–4978.9 µg/g extract), propelargonidin dimer (*Rt* = 5.65 min) (2177.2–5209.1 µg/g extract) and procyanidin B4 (1771.8–3931.9 µg/g extract) are the most abundant flavan-3-ols in leaves, whereas in barks, procyanidin B2 (378.5–4011.4 µg/g extract) and (−)-epicatechin (712.6–3827.0 µg/g extract) appear to be more abundant. This latter flavan-3-ol monomer is found in larger concentration than (+)-catechin (66.4–1592.5 µg/g extract) in all leaves and bark samples.

**Figure 2:**
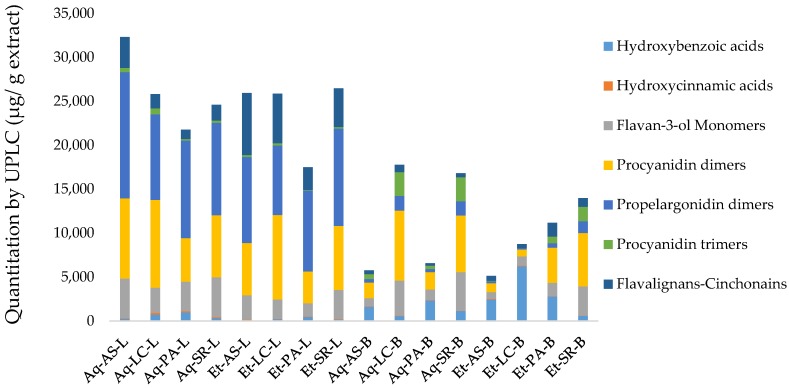
Quantification of polyphenols subclasses by Ultra Performance Liquid Chromatography coupled with Mass Spectrometry (UPLC-DAD/TQ-ESI-MS) for aqueous and ethanolic extracts of *U. tomentosa* leaves (L) and barks (B). Solvent: Aq—aqueous, Et—ethanolic; Origin: AS—Asomat; LC—Los Chiles; PA—Palacios; SR—Sarapiqui.

When considering subclasses of polyphenols (Figure 2), barks show variable contents, with procyanidin dimers as the most abundant group in Los Chiles (7971.4 µg/g of extract) aqueous extract, followed by Sarapiqui (6085.3–6464.1 µg/g of extract) in ethanolic and aqueous extracts, respectively. Los Chiles and Sarapiqui aqueous extracts exhibited the highest content of flavan-3-ols monomers (3994.4 and 4382.8 µg/g of extract, respectively), while hydroxybenzoic acids (HBA) is the third more abundant group, with the highest HBA contents in Los Chiles (6206.28 µg/g of extract) followed by Palacios (2772.6 µg/g of extract), both in ethanolic samples.

In contrast, extracts from leaves showed a more uniform subclass distribution, for instance, all extracts are particularly rich in propelargonidin dimers (7904.3–14,390.8 µg/g of extract), followed by procyanidin dimers (3593.1–9988.8 µg/g of extract) and flavalignans-cinchonains (1074.6–7058.0 µg/g of extract). 

In order to identify the influence of plant part and extraction procedure on phenolic content and distribution, a two-way ANOVA was performed in the 16 extracts. This analysis showed significant differences (*p <* 0.05) in the content of flavalignans-cinchonains in function of both factors, plant part and solvent, with leaves and ethanol rendering the highest results. On the other hand, flavan-3-ols monomers, procyanidin trimers and hydroxycinnamic acids contents were not influenced by neither factor, plant parts or solvent. In contrast, hydroxybenzoic acids, procyanidin dimers and propelargonidin dimers showed significant differences (*p <* 0.05) only when considering *U. tomentosa* part material, with hydroxybenzoic acids being more abundant in barks, while procyanidin and propelargonidin dimers being predominant in leaves. A similar trend was observed when comparing these results with previous findings reported in a detailed study on *U. tomentosa* phenolic extracts obtained through extraction and purification with organic solvents [15], which reported a higher presence of hydroxybenzoic acids in barks and a higher propelargonidin dimers content in leaves. 

In sum, our study shows that independently of the plant origin, leaves extracts exhibit high contents of phenolic compounds and more importantly high contents of proanthocyanidins derivatives, mainly propelargonidin dimers and procyanidin dimers. These compounds are linked to diverse bioactivities, for instance due to their antioxidant capacity, as shown in reports for commercial dietary products from grape [22] and cocoa [23], which suggest these *U. tomentosa* extracts’ potential for further studies. 

### 2.3. Evaluation of Antioxidant Capacity of U. tomentosa Polyphenolic Extracts

Evaluation of the antioxidant capacity of the 16 samples was performed through Oxygen Radical Absorbance Capacity (ORAC) and DPPH methods (Table 4). 

Among leaves, Asomat (12.06 µmol Trolox equivalents (TE)/mg extract) aqueous extract showed the highest antioxidant activity in ORAC methodology, followed by Los Chiles (11.57 µmol TE/mg extract) ethanolic extract, while the lowest value was obtained for Sarapiqui (6.66 µmol TE/mg extract) aqueous extract. In the case of bark samples, Sarapiqui (7.23 µmol TE/mg extract) ethanolic extract showed the highest values followed by Palacios (6.65 µmol TE/mg extract) in the same conditions, while the lowest value was found for Los Chiles (3.48 µmol TE/mg extract) aqueous extract. DPPH values for leaves followed the same trend as ORAC results, showing that Asomat aqueous extract yielded the highest value (IC_50_ = 5.23 μg/mL) followed by Los Chiles ethanolic extract (IC_50_ = 5.56 μg/mL), while Sarapiqui aqueous extract showed the lowest value (IC_50_ = 10.13 μg/mL). Referring to bark samples, DPPH values also followed a similar trend as ORAC results, with Sarapiquí ethanolic extract yielding the highest antioxidant value (IC_50_ = 6.34 μg/mL) and Los Chiles ethanolic extract showing the lowest antioxidant result (IC_50_ = 11.52 μg/mL). These values are slightly better than previous DPPH reported results [2,24] for *U. tomentosa* extracts.

To evaluate the influence of both factors, plant part and extraction solvent on ORAC and DPPH, a two-way ANOVA analysis was performed for both methods. Results indicated no significant differences for DPPH. However, ORAC antioxidant capacity was influenced by *U. tomentosa* plant part (*p <* 0.05), with leaves materials rendering better results when compared to bark samples. Considering the extraction solvent, ethanolic extracts provided slightly better results than aqueous extracts but these results were not significantly different. Literature reports on *U. tomentosa* bark and leaves extracts using aqueous and organic solvents show variable results, depending upon radical scavenging methods used, for instance some indicating better results for leaves [21] and ethanolic extraction [19] in agreement with our results. Our findings indicate both solvents yield interesting polyphenolic composition and antioxidant values, thus studies could be carried out using hydro-alcoholic mixtures to explore if results and processes could be further optimized for industrial application.

A correlation analysis was performed between both antioxidant methods and extracts phenolic contents. Results show a positive correlation between ORAC values and total phenolics determined by UPLC (*r* = 0.809, *p <* 0.05) and by the Folin–Ciocalteau method (*r* = 0.883, *p* < 0.05). Similarly, a positive correlation was found with the PRO method (*r* = 0.822, *p <* 0.05). These results are in agreement with other reports that indicate correlation between total polyphenolic content and ORAC values for several plants [25,26]. 

**Figure 3:**
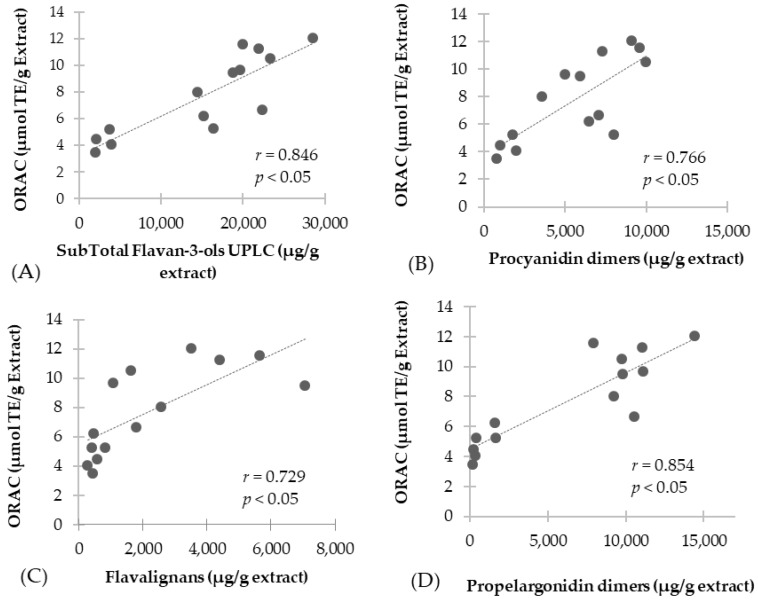
Correlation of antioxidant scavenging activity assessed by ORAC method with UPLC quantification: (**A**) Total flavan-3-ols; (**B**) Procyanidin dimers; (**C**) Flavalignans-cinchonains and (**D**) Propelargonidin dimers.

Further, Figure 3 shows the correlation analysis performed for proanthocyanidin subclasses, including total flavan-3-ols, procyanidin monomers, procyanidin dimers, propelargonidin dimers, and flavalignans-cinchonains. It should be noted the significant positive correlation with total flavan-3-ols (*r* = 0.846, *p <* 0.05) (Figure 3A), procyanidin dimers (*r* = 0.766, *p <* 0.05) (Figure 3B), flavalignans (*r* = 0.729, *p <* 0.05) (Figure 3C) and propelargonidin dimers (*r* = 0.854, *p <* 0.05) (Figure 3D), while no correlation was found for flavan-3-ol monomers. Our findings are in agreement with other studies, reporting that the antioxidant activity was lower for flavan-3-ol monomers in comparison with flavan-3-ols of higher degree of polymerization [27,28]. Also, previous studies have revealed that properlagonidin dimers from *U. tomentosa* may play a major role in conferring antioxidant capacity [17]. Furthermore, ORAC values obtained are similar to those of proanthocyanidin extracts from grape seeds [22], indicating the potential commercial value of these *U. tomentosa* extracts.

In respect to DPPH, results show correlation between total phenolics determined by UPLC (*r* = −0.596, *p <* 0.05) and antioxidant activity measured through DPPH, as well as between Folin–Ciocalteau values (*r* = −0.635, *p <* 0.05) and DPPH antioxidant values. Similarly, a correlation was found between these antioxidant results and the PRO values (*r* = −0.670, *p <* 0.05). These results are in agreement with a report on *U. tomentosa,* showing correlation between antioxidant potency of cat’s claw preparations and their protective ability against DPPH [24]. 

**Figure 4:**
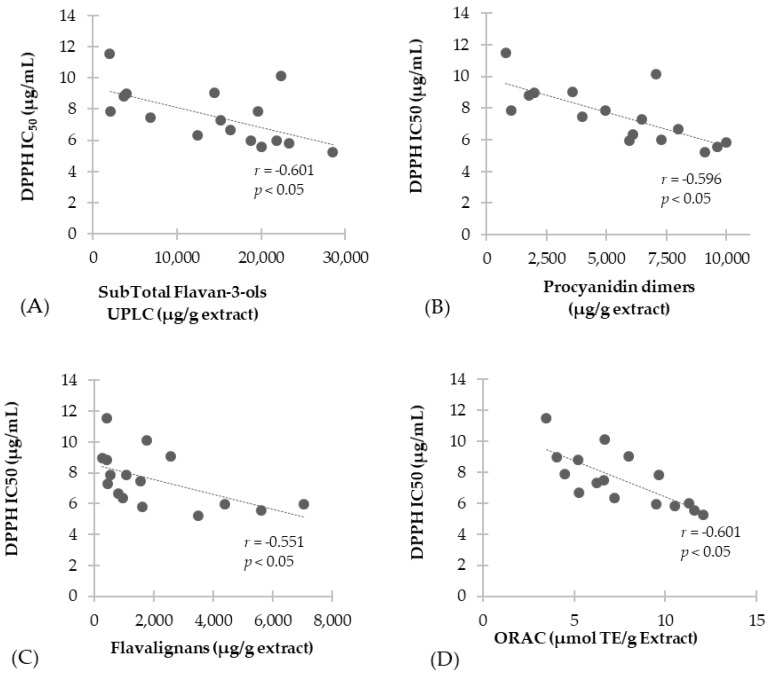
Correlation of antioxidant scavenging activity assessed by DPPH with: (**A**) Total flavan-3-ols; (**B**) Procyanidin dimers; (**C**) Flavalignans-cinchonains and (**D**) ORAC antioxidant capacity.

Further, Figure 4 shows the correlation analysis performed for proanthocyanidin subclasses. Results show a negative correlation with total flavan-3-ols (*r* = −0.601, *p <* 0.05) (Figure 4A), procyanidin dimers (*r* = −0.734, *p <* 0.05) (Figure 4B), flavalignans (*r* = −0.551, *p <* 0.05) (Figure 4C), while no correlation was found for flavan-3-ol monomers and propelargonidin dimers. Finally, Figure 4D shows negative correlation results between the antioxidant capacity evaluated through ORAC method and DPPH results (*r* = −0.735, *p <* 0.05).

### 2.4. Principal Component Analysis for Polyphenolic Extracts of U. tomentosa

To summarize the findings, a statistical Principal Component Analysis (PCA) was performed (*n* = 16) considering 32 individual phenolic compounds as well as TP, PRO, DPPH and ORAC values. Two components (PC1 and PC2) were obtained (loadings > 0.18). The first component (PC1) represented 44.9% of total variance and showed positive correlation to various proanthocyanidin compounds, including procyanidin B1, B3, B4, B7 dimers, all four propelargonidin dimers and two flavalignans-cinchonains (at *Rt* 8.99 and 9.25 min) as well as TP, PRO and ORAC values. The second component (PC2) accounted for 16.5% of the total variance and was positively correlated to four acids, namely benzoic acid, 4-hydroxybenzoic acid, ferulic acid and isoferulic acid.

**Figure 5:**
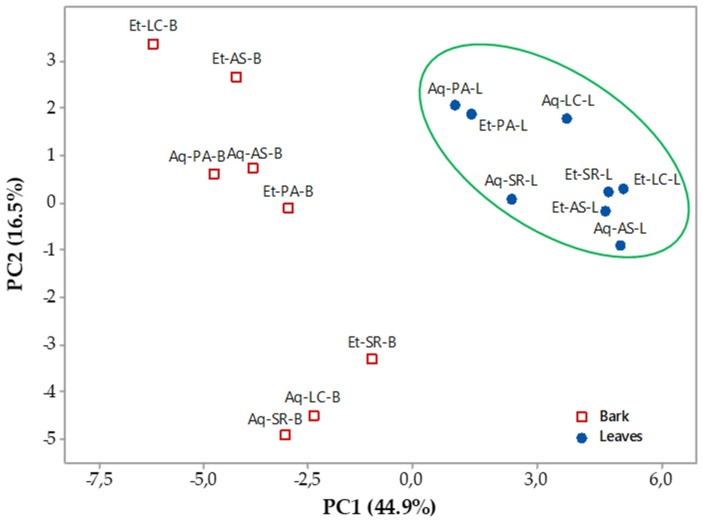
Plane defined by two first principal components (PC1 and PC2) resulting from the Principal component analysis (PCA) of individualized phenolic composition of *U. tomentosa* phenolic extracts (*n* = 16). Extraction: Aq—Aqueous, Et—Ethanolic. Origin: AS—Asomat, LC—Los Chiles, PA—Palacios, SR—Sarapiqui. Plant part: B—Bark, L—Leaves.

As illustrated in the plane represented by the two components (Figure 5), bark samples showed low scores in PC1, representing their lower values for these variables, and they are distributed along the second component (PC2) indicating high variability in the contents of the hydroxybenzoic and hydroxycinnamic acids previously cited. In contrast, leaves samples are grouped in the higher score in PC1 with larger content of polyphenols (TP), proanthocyanidin (PRO) and ORAC capacity, as well as all four propelargonidin dimers, procyanidin B1, B3, B4, B7 dimers and the two flavalignans-cinchonains derivatives mentioned above, independently of the extraction procedure.

In sum, our results, using solvents and process adequate for the development of commercial products for human consumption, clearly indicate that leaves are the most valuable sources of functional polyphenols. Even though most of the commercial preparations are derived from bark material, our findings suggest that leaves represent a more valuable source of proanthocyanidins linked to important antioxidant activity and other potential bioactivities [16,17,22].

## 3. Materials and Methods

### 3.1. Plant Material, Chemicals and Reagents

*Uncaria tomentosa* samples were collected from different places in Costa Rica: Asomat (El Amparo) and Los Chiles (northern part), as well as Palacios (Aprolece) and Sarapiqui (Caribbean part). Vouchers for all plants are deposited in the Costa Rican National Herbarium, under series no. AQ2953, AQ3331, AQ3332 and AQ3510, respectively. The plant material studied consisted of bark (B) and leaves (L) for each location. The material was separated and dried in a stove at 40 °C, being turned over every 24 h for a week until totally dry. The dried material was then ground and preserved in plastic recipients. Solvents such as ethanol, methanol, and acetonitrile were purchased from Baker (Center Valley, PA, USA). Reagents such as AAPH (2,2-azobis(2-amidinopropane) dihydrochloride), sodium molibdate, gallic acid, Trolox (6-hydroxy-2,5,7,8-tetramethylchroman-2-carboxylic acid), fluorescein, and sodium tungstate were provided by Sigma-Aldrich (St. Louis, MO, USA).

### 3.2. Extraction of Phenolic Compounds from Bark and Leaves of U. tomentosa

The ground dried material from *U. tomentosa* bark and leaves (*n* = 8) was extracted (0.05 g/mL) separately in ethanol at 25 °C during 24 h. Afterwards the solvent was removed by filtration and the extraction process was repeated three times under the same conditions. The filtrates were concentrated to dryness using a Buchi™ 215 (Flawil, Switzerland) rotavapor, and the ethanolic extracts were preserved at −20 °C until extraction. In turn, to obtain the aqueous extracts, each ground dried material from bark and leaves (*n* = 8) was extracted (0.05 g/mL) in distilled water, under stirring and heating until reaching 80 °C. After cooling at 25 °C, the solvent was removed by filtration and the extraction procedure was repeated once. The filtrates were freeze-dried in a Free Zone −105 °C, 4.5 L, Cascade Benchtop Freeze Dry System (Labconco, Kansas, MO, USA), and the freeze-dried extracts were preserved at −20 °C until extraction. 

### 3.3. Determination of Total Phenolic Content

Total polyphenolic contents were evaluated through a variation of the Singleton and Rossi method, as previously described [15], using the Folin–Ciocalteu (FC) reagent, which consists of a mixture of phosphomolybdic and phosphotungstic acids. Briefly, 0.5 mL of a dissolution from each *U. tomentosa* extract in MeOH (0.1% HCl) was mixed with FC reagent (0.5 mL) and 10 mL of Na_2_CO_3_ (7.5%). Water was added to complete 25 mL. A similar process was followed to prepare the blank, using 0.5 mL of MeOH (0.1% HCl) instead of extract. Both, extract and blank mixtures were left standing in the dark for 1 h, and afterwards absorbance was measured at 750 nm. Values of total polyphenolic content were obtained by extrapolating absorbances in a gallic acid calibration curve. Analyses were performed in triplicate. Values are expressed as mg gallic acid equivalents (GAE)/g of *U. tomentosa* extract. 

### 3.4. Total Proanthocyanidin Determination

Evaluation of total proanthocyanidin contents was performed through a variation of the Bate–Smith method, consisting of C–C interflavanic bond oxidative cleavage, as described early [16]. Briefly, 0.2 mL of a dissolution from each *U. tomentosa* extract was mixed with 20 mL of butanol/HCl (50:50) and 0.54 mM FeSO_4_. The mixture was incubated at 90 °C for 1 h, and then left cooling to 25 °C. Afterwards, butanol-HCl mixture was added to complete 25 mL. A similar process was followed to prepare the blank, but without heating. The absorbance of each *U. tomentosa* extract mixture was measured at 550 nm against the blank. Values of total proanthocyanidin content were obtained by extrapolating absorbances in a cyanidin chloride calibration curve. Values are expresses as mg of cyanidin chloride equivalents/g of *U. tomentosa* extract.

### 3.5. Analysis of Phenolic Compounds by UPLC-DAD-ESI-TQ MS

To analyze the phenolic composition of *U. tomentosa* bark and leaves extracts, an UPLC-DAD-ESI-TQ MS system was used, consisting of an UPLC coupled to an Acquity PDA eλ photodiode array detector (DAD), as well as an Acquity tandem quadrupole (TQD) mass spectrometer equipped with Z-spray electrospray interface (Waters, Milford, MA, USA) *U. tomentosa* extracts were dissolved in acetonitrile:H_2_O (1:4), for separation on a Waters^®^ BEH C18 column (2.1 × 100 mm; 1.7 μm) at 40 °C. The flow rate was 0.5 mL/min and the eluent system consisted of a gradient of solvent A—water:acetic acid (98:2, *v*/*v*)—and B—acetonitrile:acetic acid (98:2, *v*/*v*)—in the following order [15]: 0–1.5 min: 0.1% B, 1.5–11.17 min: 0.1–16.3% B, 11.17–11.5 min: 16.3–18.4% B, 11.5–14 min: 18.4% B, 14–14.1 min: 18.4–99.9% B, 14.1–15.5 min: 99.9% B, 15.5–15.6 min: 0.1% B, 15.6–18 min: 0.1% B. DAD was functioning at 250–420 nm, 1.2 nm resolution and at a 20 point/s rate. ESI was operated in negative mode and ESI parameters comprised: source temperature of 130 °C; capillary voltage of 3 kV; desolvation temperature of 400 °C; cone and desolvation gas (N_2_) with flow rates of 60 L/h and 750 L/h respectively. 

Data were collected in the MRM mode, with specific transitions of parent and product ions for each compound, whereas external calibration curves were used. Main transitions corresponded to flavan-3-ol monomers (+)-catechin and (−)-epicatechin) at *m*/*z* 289/245, to dimers of procyanidins at *m*/*z* 577/289 and of propelargonidins at *m*/*z* 561/289. Also, transitions for procyanidin trimers at *m*/*z* 865/577), and finally for flavanolignans-cinchonains at *m*/*z* 451/341. For optimizations, mass detector parameters and calibration curves, commercial standards were used for both flavan-3-ol monomers [(−) epicatechin and (+)-catechin] and two procyanidin dimers, namely B1 [(−)-epicatechin-(4β → 8)-(+)-catechin] and B2 [(−)-epicatechin-(4β → 8)-(−)-epicatechin], as well as for procyanidin trimer C1 [(−)-epicatechin-(4β → 8)-(−)-epicatechin-(4β → 8)-(−)-epicatechin]. For other procyanidin structures such as dimer B3 [(+)-catechin-(4α → 8)-(+)-catechin], B5 [(−)-epicatechin-(4β → 6)-(−)-epicatechin], and B7 [(−)-epicatechin-(4β → 6)-(+)-catechin] work was carried out with standards isolated from other plants and with confirmation by MS/MS spectrum. This method was also applied for procyanidin trimer T2 [(−)-epicatechin-(4β → 8)-(−)-epicatechin-(4β → 8)-(+)-catechin]. To confirm the structure of flavanolignans and propelargonidins, because of the absence of commercial standards, MS/MS spectrum was achieved for molecular ions at *m*/*z* 451 and *m*/*z* 561 respectively. In respect to quantification for these two types of molecules, this was carried out on the calibration curve of (−)-epicatechin and that of procyanidin dimer B1 respectively. Finally, calibration curve of procyanidin B1 was used for quantification of procyanidin dimers B2 and B3. The limit of quantification (LOQ) and the limit of detection (LOD) of the different standards are published elsewhere [29,30]. The analyses were carried out in triplicate.

### 3.6. Oxygen Radical Absorbance Capacity (ORAC)

The radical scavenging activity of the *U. tomentosa* phenolic extracts was evaluated through the ORAC method [31]. Briefly, 0.05 g of each *U. tomentosa* leaves and bark extracts were dissolved in 10 mL of methanol/HCl (1000:1, *v*/*v*), then centrifuged (3024 *g*, 5 min, 5 °C) and filtered (0.45 µm). The reaction was performed at 37 °C in 75 mM phosphate buffer (pH 7.4), whereas the final reaction mixture (200 µL) comprised fluorescein (70 nM, used as a fluorescence probe), antioxidant (Trolox (1–8 µM) or *U. tomentosa* extract (at different concentrations)) and AAPH (12 mM). AAPH and Trolox solutions were freshly prepared while a fluorescein stock solution (1.17 mM) served to prepare dilutions using 75 mM phosphate buffer (pH 7.4). Black 96-well untreated microplates (Nunc, Denmark) were used for fluorescence determinations in a Polarstar Galaxy plate reader with 520-P emission and 485-P excitation filters, as well as a Fluorstar Galaxy version v.4.11-0 software (BMG Labtechnologies GmbH, Offenburg, Germany). The 96-well microplate was automatically shaken before the first fluorescence reading and afterwards readings were recorded every minute for 98 min. The curve of the blank (no antioxidant) served to normalize the fluorescence measurements. The area under the decay curve (AUC) was calculated from the fluorescence normalized curve, and each sample net AUC was calculated using the formula: Net AUC = AUC_antioxidant_ − AUC_blank_. Afterwards, the regression equation between the antioxidant concentration and the net AUC was estimated. The actual ORAC value for each sample was calculated by dividing the slope of the later equation by the slope of the Trolox equation attained for the same assay. ORAC values were expressed as mmol of Trolox equivalents (TE)/g of *U. tonentosa* extract. Besides each reaction mixture being performed in duplicate, three independent runs were carried out for each sample.

### 3.7. DPPH Radical-Scavenging Activity

To assess the radical scavenging activity through DPPH method, a 0.25 mM solution of 2,2-diphenyl-1-picrylhidrazyl (DPPH) was prepared in methanol. Then, 0.5 mL of this solution were mixed with 1 mL of polyphenolic samples at different concentrations and the mixture was incubated at 25 °C in the dark for 30 min. Blanks were prepared using 1 mL of samples but using 0.5 mL of methanol instead of DPPH. Finally, DPPH absorbance was measured at 517 nm. Each sample was analyzed in three independent assays. Curves of absorbance vs. concentration were plotted to obtain IC_50_, which is the concentration sample needed to get 50% of radical-scavenging activity. 

### 3.8. Statistical Analysis

Two-way analyses of variance (ANOVA) were applied to subtotals of phenolic subclasses in order to determine differences in quantitative values in respect to both factors, part of plant and extraction solvents used. On the other hand, in order to evaluate if the total phenolic contents (TP, PRO) and the phenolic subclasses measured by UPLC-DAD/TQ-ESI-MS contribute to the antioxidant activity, a Pearson correlation analysis was carried out between such variables and ORAC values. Finally, a Principal component analysis (PCA) was applied to summarize the data from the leaves and bark extracts (*n* = 16) from *U. tomentosa* taking in consideration 32 individual phenolic contents, TP, PRO, DPPH and ORAC, using the above mentioned Statistical program. 

## 4. Conclusions

This paper describes successful methods to obtain enriched polyphenolic extracts of *U. tomentosa* bark and leaves with solvents adequate for human consumption. Using advanced analytical techniques such as UPLC-DAD/TQ-ESI-MS, results showed selective distribution of 32 non-flavonoid and flavonoid phenolics among the different samples, proanthocyanidins being predominant in leaves, independently of their origin or solvent used. Among proanthocyanidins, propelargonidin dimers are characteristic marker compounds in leaves, showing significant correlation with ORAC and DPPH antioxidant activity. PCA analysis also revealed that the phenolic composition of the leaves tends to be less variable than that of barks, also suggesting that leaves constitute a more homogenous material for extraction procedures like the ones used in this study. While most of products already commercialized are pulverized bark material, our results suggest that leaves constitute the part of *U. tomentosa* more suitable for use in the elaboration of standardize phenolic extracts with potential applications in the nutraceutical industry. 

## Figures and Tables

**Table 1:** Extraction yield, total phenolic (TP) and flavan-3-ols (PRO) for aqueous and ethanolic extracts from *U. tomentosa*.

Samples	Extraction Yield (%) ^1^	TP (mg/g) ^2^	PRO (mg/g) ^3^
**Aqueous Extracts**			
**Leaves**			
Asomat (AS)	4.95	335.2 ± 6.6	255.6 ± 6.5
Los Chiles (LC)	4.22	385.6 ± 1.1	304.9 ± 4.0
Palacios (PA)	3.95	311.0 ± 0.4	201.9 ± 2.6
Sarapiqui (SR)	5.45	218.2 ± 5.3	152.2 ± 2.9
**Bark**			
Asomat (AS)	1.93	236.0 ± 5.8	166.4 ± 2.3
Los Chiles (LC)	1.74	177.5 ± 1.6	108.4 ± 3.4
Palacios (PA)	1.71	138.2 ± 4.5	69.8 ± 1.9
Sarapiqui (SR)	2.65	201.5 ± 3.5	114.6 ± 2.2
**Ethanolic extracts**			
**Leaves**			
Asomat (AS)	5.99	362.4 ± 5.5	300.5 ± 6.0
Los Chiles (LC)	5.39	387.6 ± 3.0	298.6 ± 8.4
Palacios (PA)	6.12	340.4 ± 3.7	260.3 ± 3.7
Sarapiqui (SR)	6.03	342.1 ± 2.8	281.5 ±4.8
**Bark**			
Asomat (AS)	2.88	295.2 ± 3.1	198.3 ± 4.5
Los Chiles (LC)	1.26	196.2 ± 4.5	88.2 ± 3.3
Palacios (PA)	3.17	279.8 ± 6.2	194.8 ± 5.5
Sarapiqui (SR)	2.86	321.0 ± 3.6	216.7 ± 5.3

^1^ g of extract/g of dry material expressed as %; ^2^ mg of gallic acid equivalent/g extract; ^3^ mg cyanidin chloride equivalents/g extract.

**Table 2:** Phenolic composition of aqueous extracts from bark and leaves of *U. tomentosa*.

		Leaves Extracts				Bark Extracts			
Compound	AS		LC	PA	SR	AS	LC	PA	SR
Concentration (µg/g Extract)									
*Hydroxybenzoic acids*									
Benzoic acid	71.2 ± 6.9		50.3 ± 0.8	225.8 ± 7.6	120.0 ± 9.8	343.0 ± 23.2	60.3 ± 5.8	1181.6 ± 89.0	13.0 ± 0.5
Salicylic acid	27.7 ± 2.3		22.9 ± 0.4	24.7 ± 1.1	29.0 ± 1.9	20.1 ± 1.8	56.4 ± 0.9	105 ± 6.9	197.6 ± 1.4
4-hydroxybenzoic acid	35.2 ± 1.1		143.6 ± 3.2	200.0 ± 4.7	41.1 ± 1.1	113.7 ± 7.7	22.7 ± 1.1	164.7 ± 6.6	37.6 ± 1.9
Protocatechuic acid	60.5 ± 1.4		323.0 ± 5.5	495.5 ± 13.2	120.5 ± 6.9	983.8 ± 61.6	292.2 ± 9.6	696.5 ± 52.3	808.0 ± 18.7
Gallic acid	47.4 ± 1.2		188.5 ± 5.7	19.5 ± 1.4	10.7 ± 0.9	42.6 ± 3.3	25.3 ± 1.3	27.9 ± 2.9	28.3 ± 0.4
Vainillinic acid	5.8 ± 0.1		9.5 ± 0.9	10.7 ± 0.3	4.8 ± 0.1	94.8 ± 6.6	84.8 ± 8.3	136.7 ± 7.5	45.2 ± 2.3
Syringic acid	3.4 ± 0.0		6.9 ± 0.1	6.8 ± 0.1	4.4 ± 0.2	20.5 ± 1.0	26.8 ± 1.5	21.9 ± 0.5	12.7 ± 0.1
*∑ Hydroxybenzoic acids*	251.3		744.8	983.0	330.3	1618.5	568.5	2334.3	1142.5
*Hydroxycinnamic acids*									
*p*-cumaric acid	11.2 ± 1.1		42.9 ± 3.2	18.1 ± 4.7	37.5 ± 1.1	5.5 ± 7.7	5.2 ± 1.1	8.6 ± 6.6	2.2 ± 1.9
Caffeic acid	13.9 ± 0.7		91.4 ± 1.4	38.4 ± 0.9	15.3 ± 0.5	12.7 ± 0.6	15.9 ± 1.4	14.4 ± 1.3	7.3 ± 0.6
Ferulic acid	22.2 ± 0.7		46.1 ± 3.3	59.4 ± 2.0	31.2 ± 1.6	22.7 ± 1.1	12.2 ± 0.6	11.0 ± 0.5	14.3 ± 1.0
Isoferulic acid	15.4 ± 0.2		7.8 ± 0.7	12.0 ± 0.3	10.9 ± 1.0	nd	nd	nd	nd
*∑ Hydroxycinnamic acids*	62.7		188.3	128.0	94.9	40.9	33.2	34.1	23.8
*Flavan-3-ols: monomers*									
(+)-Catechin	1170.3 ± 30.5		947.2 ± 39.7	1181.0 ± 11.7	1592.5 ± 32.2	112.4 ± 2.8	691.5 ± 24.8	185.3 ± 18.4	555.8 ± 26.2
(−)-Epicatechin	3346.5 ± 46.6		1901.7 ± 39.6	2179.4 ± 68.8	2954.5 ± 73.0	835.1 ± 27.8	3302.9 ± 194.2	1032.9 ± 19.3	3827.0 ± 76.6
*∑ Monomers*	4516.8		2848.8	3360.4	4547.0	947.4	3994.4	1218.3	4382.8
*Flavan-3-ols: procyanidin dimers*									
Procyanidin B1	1233.6 ± 39.0		1536.6 ± 16.0	829.4 ± 16.3	887.1 ± 19.6	68.2 ± 1.8	587.2 ± 25.5	105.4 ± 10.8	327.4 ± 10.1
Procyanidin B2	2415.6 ± 35.6		1651.0 ± 26.9	1085.5 ± 24.9	1291.9 ± 29.8	1085.3 ± 20.8	4011.4 ± 297.6	1061.2 ± 78.3	3777.0 ± 68.0
Procyanidin B3	1119.1 ± 32.0		2417.8 ± 34.3	893.1 ± 36.3	1383.2 ± 32.7	53.2 ± 1.7	350.3 ± 17.5	70.2 ± 2.3	158.8 ± 9.5
Procyanidin B4	3642.9 ± 96.5		3888.7 ± 79.0	1886.0 ± 57.9	2979.5 ± 68.7	479.8 ± 5.5	2720.8 ± 159.0	681.5 ± 48.6	1715.3 ± 5.2
Procyanidin B5	317.1 ± 4.0		159.5 ± 7.4	137.6 ± 8.2	191.0 ± 7.3	72.6 ± 2.7	301.8 ± 18.9	50.4 ± 3.6	448.3 ± 12.2
Procyanidin B7	158.8 ± 12.2		164.2 ± 10.1	47.3 ± 2.7	118.4 ± 2.1	nd	nd	nd	nd
Procyanidin B (5.47 min)	154.8 ± 11.0		171.0 ± 12.9	63.2 ± 1.1	116.5 ± 6.7	nd	nd	nd	nd
Procyanidin B (9.27 min)	68.1 ± 2.4		nd	20.2 ± 1.4	79.0 ± 6.4	nd	nd	nd	37.3 ± 1.2
*∑ Procyanidin dimers*	9110.0		9988.8	4962.2	7046.6	1759.0	7971.4	1968.7	6464.1
*Flavan-3-ols: propelargonidin dimers*									
Propelargonidin dimer (4.43 min)	4978.9 ± 161.5		4125.9 ± 84.3	4689.4 ± 185.5	4477.4 ± 235.3	22.3 ± 1.6	126.2 ± 6.6	46.8 ± 2.7	115.3 ± 2.1
Propelargonidin dimer (5.01 min)	3369.6 ± 102.6		2661.0 ± 39.5	3179.5 ± 23.3	2981.4 ± 52.4	118.3 ± 4.1	346.7 ± 15.7	94.2 ± 9.0	257.0 ± 4.7
Propelargonidin dimer (5.65 min)	5209.1 ± 59.9		2602.3 ± 23.3	2935.9 ± 60.4	2748.8 ± 69.6	238.5 ± 5.8	1135.2 ± 71.3	213.2 ± 19.7	1153.2 ± 13.6
Propelargonidin dimer (9.27 min)	833.2 ± 5.1		352.8 ± 2.8	298.9 ± 24.8	339.6 ± 3.1	17.6 ± 1.0	56.8 ± 2.7	9.2 ± 0.9	83.2 ± 4.5
*∑ Propelargonidin dimers*	14,390.8		9742.1	11,103.7	10,547.3	396.6	1664.9	363.3	1608.7
*Flavan-3-ols: procyanidin trimers*									
Trimer T2	nd		104.6 ± 11.3	nd	nd	nd	235.1 ± 25.2	nd	nd
Procyanidin C1	303.7 ± 9.0		190.7 ± 6.3	64.3 ± 0.6	106.3 ± 3.9	480.8 ± 45.4	2006.3 ± 169.3	323.2 ± 2.1	2148.3 ± 70.4
Trimer B (5.78 min)	176.6 ± 13.9		395.7 ± 15.4	101.3 ± 6.7	145.1 ± 8.5	86.6 ± 3.8	473.6 ± 16.9	54.5 ± 3.8	585.0 ± 13.9
*∑ Procyanidin trimers*	480.3		691.0	165.6	251.4	567.4	2715.1	377.7	2733.4
*Flavalignans*									
Cinchonain (7.37 min)	937.8 ± 21.5		325.4 ± 18.0	258.3 ± 11.3	354.8 ± 25.8	183.4 ± 6.9	391.8 ± 8.6	107.2 ± 4.2	213.1 ± 10.4
Cinchonain (9.05 min)	786.9 ± 9.8		420.4 ± 5.3	249.6 ± 20.9	522.8 ± 19.8	18.9 ± 1.1	49.5 ± 2.8	22.5 ± 1.2	15.3 ± 0.3
Cinchonain (9.30 min)	977.6 ± 20.8		565.4 ± 17.4	335.5 ± 16.7	570.8 ± 20.2	24.7 ± 0.6	55.7 ± 2.6	24.0 ± 1.2	21.4 ± 1.1
Cinchonain (12.27 min)	809.3 ± 11.3		301.3 ± 7.4	231.2 ± 11.9	337.9 ± 19.1	189.3 ± 2.6	325.7 ± 11.9	115.6 ± 9.3	207.9 ± 7.3
*∑ Flavalignans*	3511.6		1612.4	1074.6	1786.3	416.3	822.7	269.4	457.7

AS—Asomat; LC—Los Chiles; PA—Palacios; SR—Sarapiquí; nd—not detected.

**Table 3:** Phenolic composition of ethanolic extracts from bark and leaves of *U. tomentosa*.

	Leaves Extracts				Bark Extracts			
Compound	AS	LC	PA	SR	AS	LC	PA	SR
Concentration (µg/g Extract)								
*Hydroxybenzoic acids*								
Benzoic acid	46.3 ± 0.2	88.9 ± 9.8	165.9 ± 8.8	64.8 ± 4.3	1053.4 ± 41.9	5102.6 ± 69.5	1567.6 ± 22.0	20.1 ± 1.4
Salicylic acid	12.0 ± 1.1	10.0 ± 0.5	12.4 ± 0.6	12.0 ± 0.6	27.6 ± 0.5	175.4 ± 2.3	100.8 ± 7.1	110.0 ± 3.3
4-hydroxybenzoic acid	19.9 ± 0.7	23.2 ± 1.3	124.3 ± 4.4	26.2 ± 1.4	203.0 ± 6.2	117.2 ± 4.6	176.2 ± 15.3	42.2 ± 2.2
Protocatechuic acid	25.7 ± 1.8	61.4 ± 1.0	118.4 ± 3.3	48.5 ± 2.8	932.9 ± 21.5	283.8 ± 4.3	742.1 ± 61.7	326.5 ± 9.2
Gallic acid	5.8 ± 0.3	10.5 ± 0.1	11.5 ± 0.6	2.8 ± 0.1	28.1 ± 1.7	32.5 ± 1.1	15.8 ± 1.1	15.4 ± 0.2
Vainillinic acid	3.5 ± 0.0	5.8 ± 0.2	12.6 ± 0.6	4.4 ± 0.1	150.3 ± 6.6	412.1 ± 16.7	147.7 ± 10.5	47.0 ± 1.8
Syringic acid	nd	3.4 ± 0.3	5.3 ± 0.2	nd	40.9 ± 2.1	82.7 ± 3.0	22.4 ± 1.3	12.5 ± 0.1
*∑ Hydroxybenzoic acids*	113.2	203.2	450.4	158.6	2436.1	6206.3	2772.6	573.8
*Hydroxycinnamic acids*								
*p*-cumaric acid	4.6 ± 0.7	8.1 ± 1.3	22.5 ± 4.4	22.1 ± 1.4	9.9 ± 6.2	7.6 ± 4.6	4.8 ± 15.3	2.6 ± 2.2
Caffeic acid	5.3 ± 0.5	7.5 ± 0.3	7.4 ± 0.4	5.4 ± 0.3	9.0 ± 0.6	7.5 ± 0.7	10.8 ± 1.0	4.5 ± 0.2
Ferulic acid	10.2 ± 1.0	17.9 ± 0.9	30.1 ± 1.9	16.7 ± 1.5	31.5 ± 1.1	32.4 ± 3.2	11.8 ± 0.9	21.7 ± 0.8
Isoferulic acid	14.4 ± 0.1	nd	7.7 ± 0.2	nd	21.0 ± 1.5	38.9 ± 4.0	nd	nd
*∑ Hydroxycinnamic acids*	34.5	33.6	67.7	44.3	71.3	86.4	27.4	28.8
*Flavan-3-ols: monomers*								
(+)-Catechin	615.1 ± 6.7	674.2 ± 16.0	412.0 ± 14.1	1173.2 ± 33.4	66.4 ± 5.5	67.7 ± 4.7	100.7 ± 8.4	181.1 ± 11.3
(−)-Epicatechin	2163.7 ± 62.3	1554.3 ± 52.9	1097.2 ± 31.8	2159.0 ± 45.3	712.6 ± 44.0	978.3 ± 38.9	1444.3 ± 142.2	3148.6 ± 64.9
*∑ Monomers*	2778.8	2228.5	1509.2	3332.2	779.0	1046.0	1545.0	3329.6
*Flavan-3-ols: procyanidin dimers*								
Procyanidin B1	621.5 ± 13.4	1114.6 ± 40.6	313.6 ± 8.4	769.6 ± 27.9	23.2 ± 0.9	21.8 ± 1.5	99.0 ± 8.2	170.5 ± 5.8
Procyanidin B2	1363.7 ± 35.7	1399.3 ± 24.3	539.1 ± 18.2	992.1 ± 36.8	527.4 ± 19.8	378.5 ± 20.2	1592.3 ± 55.9	2792.9 ± 83.7
Procyanidin B3	830.0 ± 26.6	2353.2 ± 16.1	687.6 ± 11.4	1708.5 ± 93.7	19.2 ± 1.0	14.2 ± 0.2	69.3 ± 1.6	68.3 ± 3.4
Procyanidin B4	2476.6 ± 41.9	3931.9 ± 108.9	1711.8 ± 13.6	3172.6 ± 73.6	370.9 ± 15.7	319.1 ± 17.0	1866.0 ± 86.1	2015.7 ± 10.4
Procyanidin B5	291.0 ± 6.4	268.7 ± 8.3	122.6 ± 9.9	217.2 ± 3.4	72.9 ± 2.8	53.9 ± 0.1	292.0 ± 5.6	744.6 ± 13.6
Procyanidin B7	161.4 ± 9.3	267.6 ± 6.1	85.5 ± 4.8	170.5 ± 14.5	nd	nd	nd	112.2 ± 5.5
Procyanidin B (5.47 min)	137.8 ± 9.6	263.3 ± 7.5	96.0 ± 10.6	184.3 ± 13.2	nd	nd	65.1 ± 4.3	123.1 ± 9.4
Procyanidin B (9.27 min)	53.4 ± 2.2	nd	36.8 ± 2.8	76.0 ± 9.4	nd	nd	nd	58.0 ± 3.3
*∑ Procyanidin dimers*	5935.4	9598.5	3593.1	7290.8	1013.6	787.4	3983.8	6085.3
*Flavan-3-ols: propelargonidin dimers*								
Propelargonidin dimer (4.43 min)	3239.3 ± 63.1	3067.8 ± 72.3	3644.5 ± 59.8	4660.6 ± 105.0	9.9 ± 1.0	22.0 ± 0.8	16.4 ± 2.4	68.7 ± 5.7
Propelargonidin dimer (5.01 min)	2503.6 ± 26.9	2179.3 ± 22.1	2926.1 ± 37.8	3343.7 ± 57.1	85.7 ± 6.0	58.0 ± 5.1	153.6 ± 6.4	231.7 ± 18.0
Propelargonidin dimer (5.65 min)	3253.4 ± 12.5	2177.2 ± 33.1	2178.4 ± 82.6	2480.8 ± 38.0	112.8 ± 4.3	77.6 ± 2.4	308.0 ± 13.8	895.5 ± 24.3
Propelargonidin dimer (9.27 min)	795.7 ± 30.3	480.0 ± 15.5	471.1 ± 20.5	576.8 ± 21.0	12.2 ± 1.2	12.4 ± 0.8	42.3 ± 1.3	143.8 ± 5.7
*∑ Propelargonidin dimers*	9792.0	7904.3	9220.2	11,061.9	220.6	169.9	520.2	1339.7
*Flavan-3-ols: procyanidin trimers*								
Trimer T2	nd	nd	nd	nd	nd	nd	nd	nd
Procyanidin C1	243.4 ± 25.4	260.6 ± 13.2	69.7 ± 0.4	181.2 ± 9.1	37.8 ± 0.4	nd	744.6 ± 10.8	1636.2 ± 76.2
Trimer B (5.78 min)	nd	nd	nd	nd	nd	nd	nd	nd
*∑ Procyanidin trimers*	243.4	260.6	69.7	181.2	37.8	0.0	744.6	1636.2
*Flavalignans*								
Cinchonain (7.37 min)	1723.6 ± 23.5	1011.9 ± 17.8	567.2 ± 14.4	773.0 ± 33.1	236.0 ± 15.1	170.7 ± 9.8	745.7 ± 20.4	447.7 ± 2.5
Cinchonain (9.05 min)	2010.3 ± 17.0	1816.3 ± 48.4	744.3 ± 23.4	1544.1 ± 77.1	19.7 ± 1.2	20.7 ± 2.6	63.8 ± 5.1	28.4 ± 1.4
Cinchonain (9.30 min)	1795.3 ± 31.1	1901.5 ± 32.5	773.9 ± 23.1	1358.0 ± 65.7	22.7 ± 2.0	25.8 ± 0.9	56.0 ± 2.5	34.6 ± 1.9
Cinchonain (12.27 min)	1528.8 ± 20.7	900.5 ± 14.5	486.7 ± 16.2	727.2 ± 23.3	285.3 ± 10.1	217.3 ± 12.9	710.2 ± 25.7	471.9 ± 17.3
*∑ Flavalignans*	7058.0	5630.1	2572.0	4402.1	563.7	434.4	1575.7	982.6

AS—Asomat; LC—Los Chiles; PA—Palacios; SR—Sarapiquí; nd—not detected.

**Table 4:** Oxygen Radical Absorbance Capacity (ORAC) and 2,2-diphenyl-1-picrylhidrazyl (DPPH) antioxidant activity of aqueous and ethanolic extracts from bark and leaves of *U. tomentosa.*

	ORAC (µmol TE/mg) ^1^		DPPH IC_50_ (μg/mL)	
Extraction	Leaves	Bark	Leaves	Bark
**Aqueous**				
AS	12.06 ± 0.36	5.22 ± 0.10	5.23 ± 0.02	8.83 ± 0.21
LC	10.53 ± 0.43	5.27 ± 0.15	5.81 ± 0.01	6.66 ± 0.15
PA	9.65 ± 0.44	4.07 ± 0.15	7.84 ± 0.03	8.98 ± 0.06
SR	6.66 ± 0.13	6.22 ± 0.28	10.13 ± 0.15	7.31 ± 0.16
**Ethanolic**				
AS	9.48 ± 0.40	4.47 ± 0.15	5.95 ± 0.05	7.88 ± 0.18
LC	11.57 ± 0.33	3.48 ±0.13	5.56 ± 0.10	11.52 ± 0.05
PA	8.01 ± 0.28	6.65 ± 0.26	9.05 ± 0.23	7.47 ± 0.17
SR	11.27 ± 0.51	7.23 ± 0.19	5.98 ± 0.03	6.34 ± 0.07

^1^ µmol Trolox equivalents/mg extract. Origin: AS—Asomat, LC—Los Chiles, PA—Palacios, SR—Sarapiqui.

## Supplementary Materials

The following are available online at http://www.mdpi.com/2076-3921/7/5/65/s1, Figure S1: MRM chromatograms (UPLC-DAD-ESI-TQ-MS) for flavan-3-ols in *U. tomentosa* extracts.
