# Supplementary material for: Polyphenolic Composition and Antioxidant Activity of Aqueous and Ethanolic Extracts from Uncaria tomentosa Bark and Leaves
Source: Antioxidants (Basel). 2018 May 11;7(5):65. doi: 10.3390/antiox7050065 (PMC5981251; doi:10.3390/antiox7050065)
Supplement: Supplementary file 1 [file antioxidants-07-00065-s001.pdf]

Supplementary Material

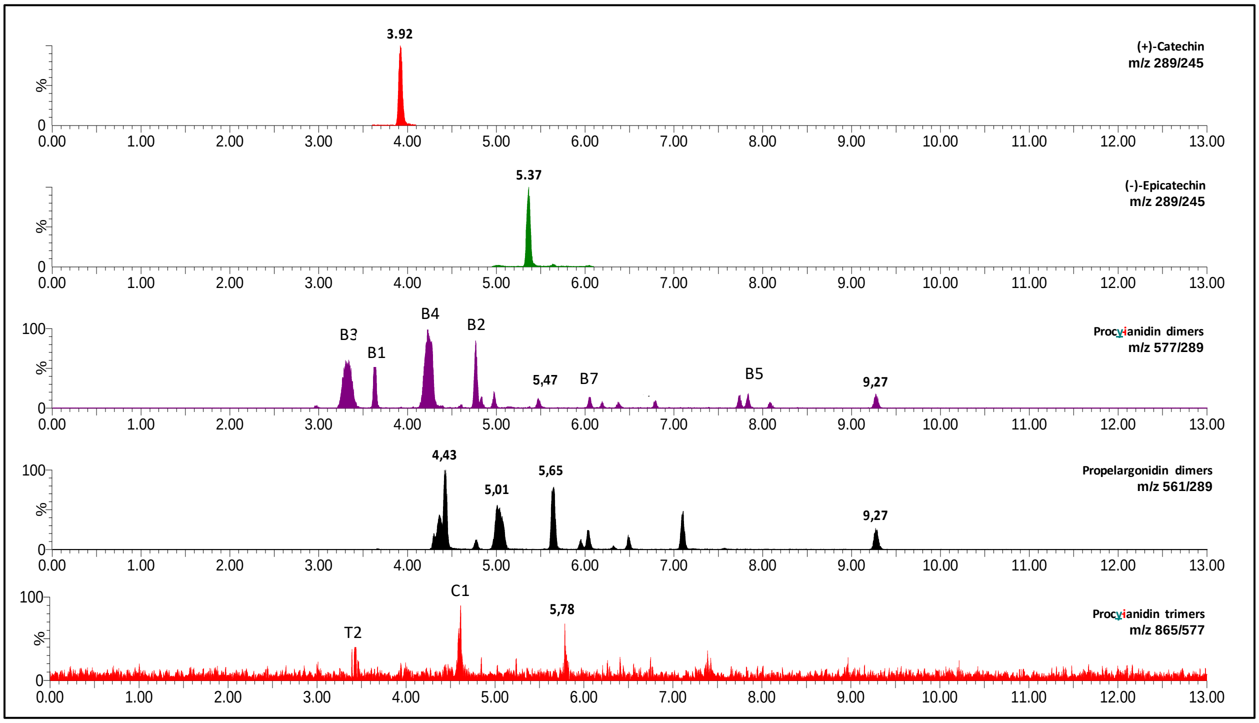

Figure S1. Multiple reaction monitoring (MRM) chromatograms (UPLC-DAD-ESI-TQ-MS) for flavan-3-ols in *U. tomentosa* extracts.
